# Macromolecular Nanocrystal Structural Analysis with Electron and X-Rays: A Comparative Review

**DOI:** 10.3390/molecules24193490

**Published:** 2019-09-26

**Authors:** Krishna P. Khakurel, Borislav Angelov, Jakob Andreasson

**Affiliations:** 1 Institute of Physics, ELI Beamlines, Academy of Sciences of the Czech Republic, Na Slovance 2, CZ-18221 Prague, Czech Republic; Borislav.Angelov@eli-beams.eu (B.A.); Jakob.Andreasson@eli-beams.eu (J.A.); 2 Department of Physics, Chalmers University of Technology, 412 96 Gothenburg, Sweden

**Keywords:** nanocrystallography, X-ray free-electron laser, electron diffraction

## Abstract

Crystallography has long been the unrivaled method that can provide the atomistic structural models of macromolecules, using either X-rays or electrons as probes. The methodology has gone through several revolutionary periods, driven by the development of new sources, detectors, and other instrumentation. Novel sources of both X-ray and electrons are constantly emerging. The increase in brightness of these sources, complemented by the advanced detection techniques, has relaxed the traditionally strict need for large, high quality, crystals. Recent reports suggest high-quality diffraction datasets from crystals as small as a few hundreds of nanometers can be routinely obtained. This has resulted in the genesis of a new field of macromolecular nanocrystal crystallography. Here we will make a brief comparative review of this growing field focusing on the use of X-rays and electrons sources.

## 1. Introduction

In the process of interrogating structure and function of molecules with probes like X-rays and electrons, it was found that putting them in a regular array amplifies the scattered signal to an extent as large as the square of the number of molecules in the array [1]. The technique for determination of the structure of protein molecules from their crystal diffraction is broadly termed as macromolecular crystallography. Conventional macromolecular crystallography needs large crystals of high quality in order to obtain diffraction maps, which can generate interpretable structures. With the emergence of new sources of both X-rays and electrons, the necessity for big crystals is in the process of being relaxed. Recently, experiments have been performed with crystals as small as a few hundred nanometers [2,3]. This opens the possibility of obtaining structures of many molecules, such as membrane proteins, for which growing high quality crystals of sufficient size has been found to be extremely challenging. The capability to obtain structures from such tiny nanocrystals has been demonstrated with both X-ray and electrons. The method of solving the structure of macromolecules with such tiny crystals has been introduced as nanocrystallography and will be the primary content of this review. 

An ideal structural method for biological macromolecules would be the one that would probe a single macromolecule and provide atomistic resolution structural information. However, despite progress and significant efforts in single particle imaging methods with X-rays [4] and electrons [5], this feat has not yet been achieved and crystallography remains unrivalled for structural determination. In crystallography, the structure obtained is the average of many identical copies of the molecules, sharing the dose requirement amongst the molecules [6]. However, arranging a large number of molecules in a regular array rarely gives a defect-free crystal. The smaller the crystal, the lesser the defect is and hence, high quality data can be obtained from small crystals [7]. This goal is largely accomplished with the emergence of nanocrystallography. In this review, we strictly define nanocrystal as the crystals which are of submicron size. Keeping crystals to this size, the number of molecules over which the signal is averaged is decreased by six orders of magnitude, or more, compared to traditional standard crystallography. The field of nanocrystallography is still in its infancy. Predictions have been made that with current generation pulsed X-rays and coherent electron beams, crystals of protein molecules with as little as few tens of unit cells in each dimension will be sufficient to obtain structural insights at high resolution [8]. The field of nanocrystallography has progressed with both electrons and X-rays. Here, we will briefly review the development in both domains. A comparison between electron and X-ray crystallography applied to nanocrystals will be made in order to introduce them as complementary techniques. Challenges present in the field will be reviewed and we will offer a glance to the future of this emerging field.

## 2. Nanocrystal X-Ray Crystallography

The origin of X-ray crystallography dates back to 1912 when Max Von Laue detailed the physics of the interaction of crystalline material with electromagnetic radiation, specifically with X-rays [9]. Since then, the method has undergone stupendous development and has become an indispensable tool in the study of physical and chemical properties of the materials. For biological sciences, it was only after Watson and Crick showed that fiber diffraction could be useful in determining the structure of DNA, that the method started to grow exponentially [10]. This was followed by the historic results from Max Perutz and John Kendrew, solving the atomistic structure of hemoglobin and myoglobin, respectively, exploiting X-ray crystal diffraction [11,12]. In the 1970s, the first series of synchrotrons appeared and this boosted the field of protein crystallography, reducing the size of the crystals necessary to get atomistic models [13]. Another significant boost was received in the 1990s when third-generation synchrotron came into operation and started delivering microbeams in routine fashion. With this development, the necessary crystal size shrunk even further into tens of micron and the method of micro-crystallography gained wide popularity in the macromolecular crystallography community. In the last decade, the powerful femtosecond X-ray lasers have caused a revolution in the X-ray community, delivering a peak power ten orders of magnitude or more higher than previously available X-ray sources [14,15,16]. In addition to many other scientific achievements, this has also resulted in a further decrease in the necessary crystal volume. Currently, submicron crystals are routinely measured at such facilities and a new field of X-ray nanocrystallography has been further developed.

Protein nanocrystallography with the synchrotron X-rays has already been discussed in the early 2000s [17,18]. In these approaches, use of nanotechnologies have been made for the growth and characterization of nanocrystals and the micro-focused beam from the synchrotron X-rays was used for data collection. The Langmuir–Blodgett nanotemplate method appeared as a valuable method in growing stable micro/nanocrystals of proteins which had not been previously crystallized [19]. 

**Figure 1:**
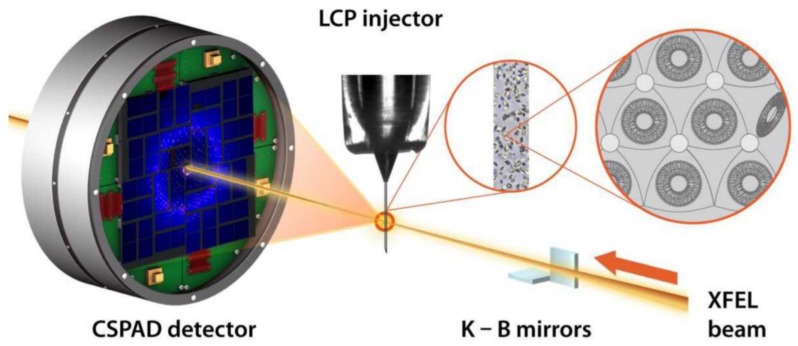
Experimental setup for serial femtosecond crystallography (SFX) data collection using an LCP injector. Microcrystals dispersed in LCP are injected as a continuous column of 20–50 μm diameter and intersected with 1.5 μm diameter pulsed XFEL beam focused by Kirkpatrick–Baez (KB) mirrors. Single pulse diffraction patterns are collected at 120 Hz using a CSPAD detector. (Adapted from Liu et al. [28]).

Although discussed theoretically already in 2000 [20], the field of X-ray nanocrystallography with ultrashort (shorter than the damage formation in the sample) pulses was experimentally verified with the seminal paper from Henry Chapman and colleagues where they shot streams of submicron-sized crystals of photosystem II with the ultrashort ultra-bright pulses of the LCLS X-ray free-electron laser [2]. In these experiments, crystals as small as ~200 nm were shot to collect diffraction data. This method of shooting streams of tiny crystals with pulses of X-ray laser is widely known as serial femtosecond crystallography (SFX) [21,22]. The initial trials resulting in the low-resolution diffraction datasets using the SFX method were soon extended to reach atomic resolution [23,24,25,26]. In vivo protein crystallization found its way to nanocrystallography with the seminal works of Koopmann et al., [27]. Further, the structure of membrane protein from nanocrystals grown in lipidic cubic phase was solved with SFX [28,29]. Johansson et al., demonstrated the usefulness of the technique to solve the structure of the photosynthetic reaction center [30] and the same year Barends et al. demonstrated the solution of a de novo protein crystal structure [31]. Demicri et al. demonstrated the extension of the method to solve structures from ribosome microcrystals [32]. Young et al. reported the structure of photosystem II and the substrate binding at room temperature [33]. Room temperature studies of bacterial phytochrome were reported by Edlund et al. [34] and the crystal structure of CO-Bound Cytochrome C oxidase was reported at room temperature [35]. The crystal structure of rhodopsin bound to Arrestin was reported by Kang et al. [36]. Utilizing the ultrashort pulse duration of the X-ray FEL, the method soon found its application in several exciting results in time-resolved studies [37,38]. Time-resolved studies were also made on Photosystem II by Kupitz et al. [39]. Structures of riboswitch intermediate states performed by mix and inject XFEL nanocrystallography were demonstrated [40]. In cellulo crystallography of alcohol oxidase in yeast cells has been performed [41] and nanocrystallography measurement of early stage synthetic malaria pigments has been reported by Dilanian et al. [42]. Interesting applications of the nanocrystallography of in vivo crystallization of Cathepsin B were performed [43]. Recent experiments on mosquito larvicide BinAB nanocrystals have also been reported [44]. Gati et al. have reported 2 Å resolution structure from native nanocrystalline granulovirus occlusion bodies [45]. Though most of these data have been collected in on-flight mode (from samples in a liquid jet), a few instances of goniometer-based data collection have also been reported for X-ray nanocrystallography [46]. Furthermore, the use of two-dimensional nanosized crystals has been reported in a few instances [47]. The X-ray nanocrystallography, so far, has been realized predominantly with the X-ray free-electron lasers and significant developments in sample delivery and data analysis have been made to support this development [48,49]. A schematic of the protein nanocrystallography with XFEL is shown in Figure 1. With the emergence of the new diffracted limited storage rings [50], supplemented by supremely efficient nano-focusing optics, we may soon be witnessing the realization of nanocrystallography at next-generation synchrotrons as well. Such a development would make X-ray nanocrystallography more accessible to a large number of users and will make an even more pronounced impact on the structural biology community.

Data treatment for X-ray serial nanocrystallography differs from the one used for traditional single crystal diffraction methods. The diffraction data are collected from many randomly oriented crystals and each diffraction pattern is considered as “stills”. Existing auto-indexing algorithms are used for finding the orientation of each crystal. The coherent nature of the source can result in the presence of the intensities from the shape transforms of the crystal around the Bragg peak. Optimized peak searching algorithms are used for such diffraction data. Due to the inhomogeneity in the crystal shape, size and orientation Monte–Carlo integration of the intensities around the Bragg peak is performed to obtain the structure factor, which can be used to obtain the electron density of the molecule of interest. A detailed description of the data treatment for XFEL nanocrystallography can be found in Kirian et al. [51]. 

## 3. Nanocrystal Electron Crystallography

The history of electron crystallography is relatively new. The endeavor of electron crystallography began with the realization by Aaron Klug that the phases can be extracted from the Fourier transform of the electron micrographs [52]. His efforts on the development of electron crystallography and the study of nucleic acid complexes were recognized with the Nobel Prize for chemistry in 1982. For protein crystals, it began only in the 1970s when Richard Henderson used it in solving the structure of bacteriorhodopsin from their naturally occurring two-dimensional crystals [53]. In 2005, the structure of two-dimensional Aquaporin was solved to a resolution of 1.9 Å [54]. The field of electron crystallography remains largely limited to two-dimensional crystals, although some instances of electron crystallography of microcrystals were reported in the mid-1970s by Dorset et al. [55]. Recent developments in electron source, electron optics, and detection techniques, as well as in software, have enabled electron crystallography of three-dimensional micro-crystals. This field is widely known as MicroED [56]. Owing to the fact that electrons scatter strongly, a crystal of submicron size is preferred to avoid the loss of information by multiple scattering effects. Structures from crystals, as small as a few hundred of nanometers, have been demonstrated with electron diffraction recently. This marks the beginning of the highly potential field of electron nanocrystallography. 

The MicroEd technique in the last few years has largely focused on methodology development. After the demonstration of electron crystallography for three dimensional crystals in 2013 by Shi et al. [57], significant progress has been made in data acquisition and treatment. Large efforts have also been made in crystal preparation, such as producing the lamella of the crystals in order to reduce the thickness of the crystals in the beam propagation direction [58,59]. The recent progress in electron crystallography has not only been facilitated by the developments in source and detection techniques. Revolutionary developments have also been made in the techniques for sample delivery to the electron beam. The flash freezing technique, commonly employed in the cryo electron microscopy system, has been recently recognized with the Nobel Prize for Chemistry in 2017 [60]. By this technique it is possible to keep the sample in the near-native condition and minimize radiation damage issues. These developments have been translated to the electron crystallography community. Many reviews on MicroEd have been written in recent times [61,62,63], where additional information can be found. Some of the recent works on MicroED include solving the structure of the toxic core of alpha-synuclein [64], building the atomic model with charges [65], solving the structures of HIV-1 Gag CTD-SP1 [66], atomic resolution structures from fragmented protein crystals [67], and solving the previously unobserved polymorph of hen egg-white lysozyme [68]. Xu et al. recently reported the solution of the structure of new protein R2lox using MicroED [69]. Recently, structures from microcrystals embedded in the lipidic cubic phase have been solved using MicroED [70].

**Figure 2:**
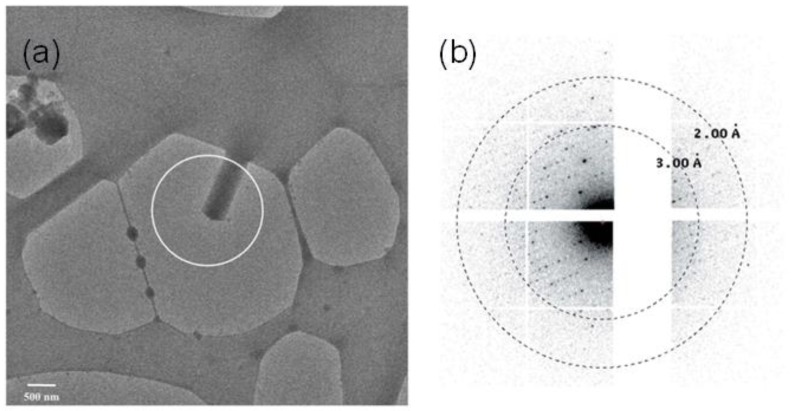
(**a**) Electron micrograph of a nanocrystal and (**b**) the diffraction from the selected area in the micrograph. (Adapted from Clabbers et al. [3]).

An ideal case for electron crystallography would be with nanocrystals, as multiple scattering events are expected to be minimized with such samples. With the use of nanocrystals, no intense sample preparation, such as ion beam milling, will be required and nanocrystallography represents a very promising niche for electron crystallography. A few instances of electron crystallography with such intrinsic nanocrystals have been reported. In 2013, Igor et al. reported the first such case [71]. Lysozyme nanocrystals with a thickness of the order of 100 nm were reported to collect diffraction maps at a resolution higher than 2 Å. In 2016, Sawaya et al. reported the first ab initio structure determined from prion nanocrystals at atomic resolution [72]. In 2017, Clabbers et al., reported electron crystallography with crystals as small as 140 nm^3^ in volume. It was reported that such crystals diffracted to 2.1 Å resolution [3]. An image of the nanocrystal and the diffraction pattern reported in the article is shown in Figure 2a,b respectively. Electron nanocrystallography has also been performed with automated diffraction tomography [73,74] and rotation electron diffraction [75]. However, these techniques, so far, have been limited to the nanocrystals of inorganic and organic materials which are relatively radiation-hard compared to the biological samples.

Data analysis in electron nanocrystallography is not straightforward. Relatively flatter Ewald’s sphere, imprecise tilt measurement, and distortion in measured intensities by the electron lens makes data analysis challenging with the existing software used for single-crystal X-ray crystallography. For the first experiments in MicroED, where the still diffraction patterns were recorded, the authors used self-developed software to perform the data reduction [76]. Lately, collection of electron diffraction in continuous rotation mode has been performed [77,78]. With this mode of operation, and making specific changes to the electron crystallography, standard software such as MOSFLM [79], DIALS [80], and XDS [81] have been reported to be used in the treatment of electron crystallography data. Following the data integration, merging, and scaling, standard crystallographic suites can be used for phasing and structure refinement. 

Crystallography with electrons is not limited to the continuous sources. Ultrafast electron sources are also under constant development [82]. Several recent works have been reported on ultrafast electron nanocrystallography of inorganic and small molecule crystals [83,84,85]. For macromolecules, a notable improvement in the source parameters, such as flux and coherence, is needed in order to realize ultrafast electron nanocrystallography.

## 4. Advantage of Nanocrystallography

Although the field of nanocrystallography has emerged in parallel with nanotechnology, some of its advantages are already obvious. The primary benefit of nanocrystallography is that it can be applied to the broad category of molecules for which high-quality crystals do not grow into sufficiently big sizes suitable for standard synchrotron-based X-ray crystallography. Such molecules could be membrane proteins, intrinsically disordered proteins, and molecular complexes. The possibility of getting atomic structures from nanocrystals also opens the application of crystallography to in vivo studies of naturally occurring nanocrystals, which is preferable for the study of the structure and function of proteins in their naturally occurring state. Nanocrystallography also opens the possibility of solving the structure of proteins, naturally occurring in trace amounts.

The field of nanocrystallography has also opened up the possibility of exploiting novel phasing options. Coherent phasing of crystals, which was envisioned by David Sayre in the early 1950s, can now be realized with the introduction of nanocrystallography, as highly brilliant coherent sources of X-rays and electrons become available [86,87]. Such phasing would relax the necessity of adding heavy atoms to the molecules during crystallization or making use of multiple wavelength measurements. Such a direct phasing method would also not need any homologous structures to solve the phase.

Smaller volume crystals are also desired for pump–probe crystallography. In a small sample, a larger fraction of the molecules can also be reached when the pump beam has limited penetration depth. This is, for example, the case when terahertz radiation is used to activate the sample. In such demanding cases of pump–probe experiments, a small sample volume ensures that the molecules in the crystals are homogeneously excited by the pump field. This allows a more accurate interpretation of the structure and function of the protein in time-resolved studies.

Protein crystals are never perfect, they usually occur in poorly aligned blocks of ordered arrangements. In crystallographic terms, they are always mosaic. In standard continuous rotation crystallography, mosaicity is supposed to present additional challenges in the structural solution and the larger the crystal size, the higher the mosaicity. This is unarguably true when seeding is done in growing crystals of suitable size to be used for single crystal X-ray diffraction. With the introduction of nanocrystallography, the mosaicity of the crystal has less of an effect on the structure solution [88]. In addition to these obvious benefits, many presently unseen benefits of nanocrystallography can be expected to appear as the field develops towards maturity.

## 5. Challenges in Nanocrystallography

There are challenging issues in nanocrystallography as well. A common issue in micro and macro crystallography is to find suitable conditions to grow protein crystals of sufficiently large size. Similar challenges persist with nanocrystallography. Apart from the actual growth of the crystal, a critical challenge arises in the screening of the crystal, as no noninvasive technique exists to observe such tiny crystals. Alignment of the nanocrystals with the micro-focused or nano-focused X-ray or electron beam presents additional challenges. The use of a large number of crystals for SFX experiments has also raised concerns about sample consumption. This concern persists, although the increase in XFEL rep rates to the MHz range [89] and the development of sample delivery systems with flow rates optimized for the different repetition rates of different X-ray sources [90], as well as concepts for online hit finding [91], are addressing these topics. Sample clogging in the jet and the possibility of mechanical damage to the crystal are some other challenges in femtosecond X-ray nanocrystallography. Additionally, growing such a large number of crystals with homogeneous sizes is a daunting task. X-ray nanocrystallography with solid support presents challenges such as reduced signal to noise ratio and modulation of the diffraction signal by the refracted signal from the support. 

With the smaller number of molecules in the beam and the trade-offs necessary to minimize the radiation damage (unless ultrashort pulses are used), the number of electrons and photons reaching the detector after interacting with the sample is often relatively small. This brings challenges to the detection techniques as all signals reaching the detector should be recorded and differentiated from the various sources of noise. With the emergence of the new field of nanocrystallography, new challenges also materialize on the side of data analysis and interpretation. In response to this, a completely new set of data analysis tools have been introduced for the X-ray free-electron laser-based nanocrystallography [92,93,94]. For electron diffraction experiments, methods to circumvent the multiple scattering have to be developed. Furthermore, the progress in the development of dynamic refinement tools has to be accelerated. Several groups have responded to these challenges and are making progress at an exciting pace [95,96,97]. A summary of the current challenges in nanocrystallography is presented in Table 1. With all these challenges addressed, the techniques will be more ready for the study of the structure and function of new proteins.

## 6. Electron Vs. X-Rays

For a long time, electron and X-ray diffraction have been seen as rivaling techniques. Both techniques are well-described with their individual merits and demerits in various earlier literature. Here we present both tools as complementary techniques for nanocrystallography. 

Compared to X-rays, electrons scatter strongly [98]. This presents both advantages and challenges with electrons. They can scatter efficiently from a small volume of molecules. However, for the size of the sample from which high resolution structural information can be obtained, the data may still be affected by multiple scattering effects. Radiation damage is another issue that can be especially serious with electrons and it can lead to low resolution or false interpretation of the structures. Several key technologies have been introduced in the past decades to solve this issue. However, the employment of such techniques will prevent room temperature measurements, which are important to obtain the physiologically relevant structures. Although the development of ultrafast electron sources is in a state of rapid progress, the field of electron nanocrystallography is still largely limited to static studies or studies of structures relevant to slow phenomena. 

Due to the fact that the electron interacts with the Coulomb potential in the atom, it has proven to be useful in locating the position of hydrogen atoms and other ions in the structure more precisely. This is otherwise extremely challenging with the X-rays. Furthermore, the availability of instruments for electron nanocrystallography in university scale laboratories makes it a more readily accessible resource than the X-ray free-electron lasers used for X-ray nanocrystallography.

X-ray nanocrystallography, in present days is mainly realized with X-ray free-electron laser. These sources use extremely short pulses with very high intensity. In the SFX experiment the nanocrystals are ionized and destroyed after the scattered field leaves the crystal. In this method, generally termed “diffraction before destruction” the effects of radiation damage on the sample structure are limited as long as the X-ray pulse duration is sufficiently short. [16,99]. The use of ultrashort pulses makes it ideal for time-resolved studies, but the large number of shots needed for structural determination comes with the additional challenges in data management and treatment. Statistical scaling of intensities from the partial reflections in the data may lead to misinterpretations of the data. On the good side, the measurements can be done at room temperature. This makes it more likely to obtain physiologically relevant structures. All in all, electron and X-ray nanocrystallography have to be taken as complementary tools to take full advantage of the growing field of nanocrystallography. A summary of the comparison of the state of the art X-ray and electron nanocrystallography is presented in Table 2.

## 7. Future Prospects in Nanocrystallography

Nanocrystallography, both with electrons and X-rays, is a field in its infancy and further developments in crystal growth, sample screening and delivery, data collection, and data analysis have to be made before it becomes a routine method for solving the structure of proteins. Nanocrystals are suitable to study the static and dynamic structure of the molecules that are difficult to grow into larger crystals. Progress in screening techniques has to be made in order to ensure that nanocrystallography becomes a standard tool in structural biology. Work on data analysis in nanocrystallography can result in novel phasing methods that could solve issues with several protein crystals that cannot otherwise be phased. Pulsed X-ray and electron sources offer new possibilities for time-resolved studies when combined with nanocrystallography. This development is presently well underway at X-ray FELs. New laser-driven X-ray and electron sources operating in the femtosecond pulsed regime, like those developed for user operation at the ELI Beamlines facility, will offer additional capacities for pump–probe experiments at a high level of synchronization and control. Overall, the field of nanocrystallography is expanding to complement other biophysical techniques in the quest to increase our knowledge of the macromolecules. 

## Figures and Tables

**Table 1:** Challenges in nanocrystallography.

	Nanocrystallography
Screening, crystal identification	Need for development of less invasive tools for screening and crystal identification.
Detector Requirements	The photons reaching detector are sparse with small crystals. Necessity of sample background reduction.
Radiation damage	Methods to minimize radiation damage in nanocrystals with continuous and pulsed X-ray and electron sources have to be found. One approach is to use ultrashort pulses
Data analysis	Challenging due to datasets from multiple crystals in unknown orientation.

**Table 2:** Comparison of X-ray and electron nanocrystallography.

	X-Ray Nanocrystallography	Electron Nanocrystallography
Source/accessibility	Large Facilities/Less accessible	Laboratory Sources/Frequent accessibility
Sample consumption	Large to moderate with serial crystallography	Less
Radiation damage	Minimal with X-ray FEL	Large and limits the highest attainable resolution. However, can be overcome by merging a large number of low dose datasets
Room temperature studies	Possible with current technologies	Not possible with current technologies
Ultrafast Time resolved studies	Possible	Not possible
Sensitivity to ions and H-atom	Less sensitive	Highly sensitive
